# Giant Chloroviruses: Five Easy Questions

**DOI:** 10.1371/journal.ppat.1005751

**Published:** 2016-08-18

**Authors:** James L. Van Etten, David D. Dunigan

**Affiliations:** Department of Plant Pathology and Nebraska Center for Virology, University of Nebraska, Lincoln, Nebraska; University of Florida, UNITED STATES

## What are the Chloroviruses?

**Fig 1:**
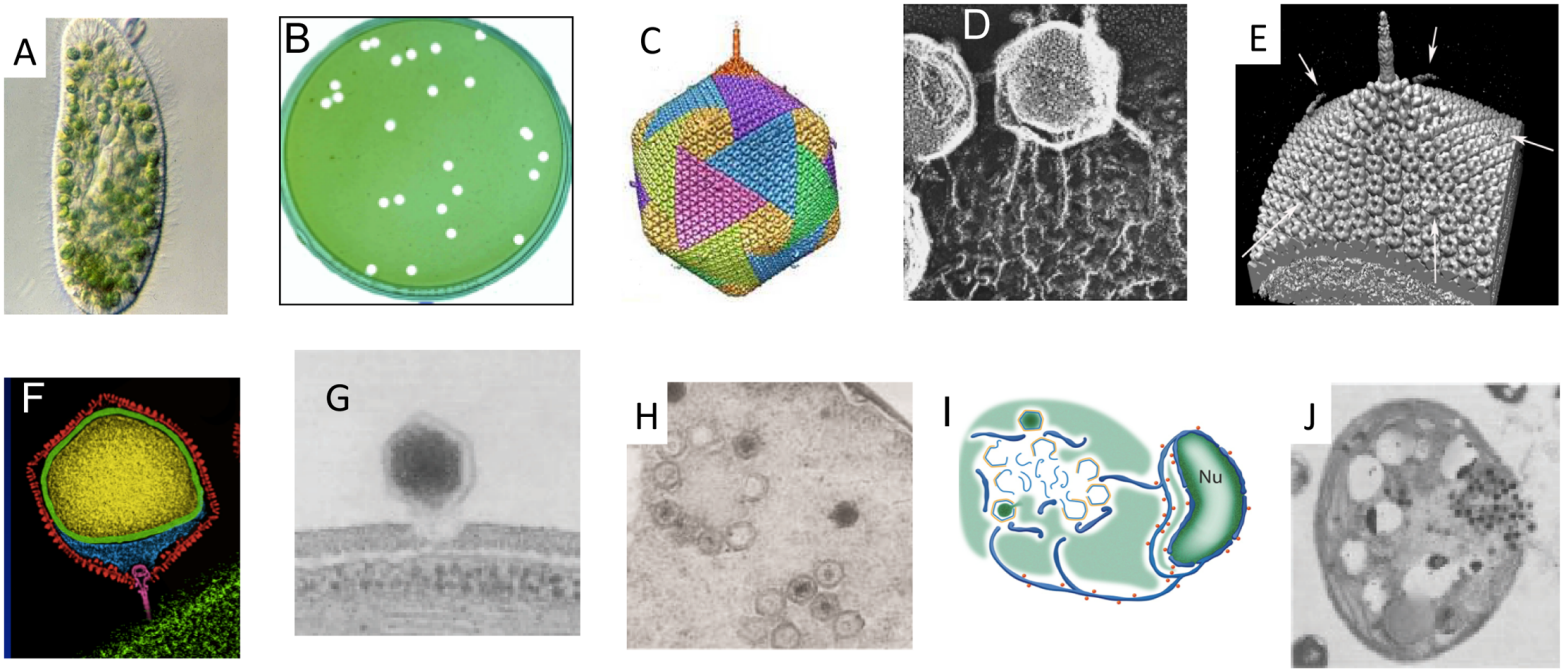
Chlorella cells and chlorovirus *Paramecium bursaria* chlorella virus (PBCV-1). (**A**) *Paramecium bursaria* and its symbiotic chlorella cells. (**B**) Plaques formed by PBCV-1 on a lawn of *Chlorella variabilis*. (**C**) Five-fold averaged cryo-electron micrograph of PBCV-1 reveals a long narrow cylindrical spike structure at one vertex and fibers extending from one unique capsomer per trisymmetron. (**D**) PBCV-1 attached to the cell wall as viewed by the quick-freeze, deep etch procedure. Note the virions attached to the wall by fibers. (**E**) Surface view of the PBCV-1 spike structure and fibers. (**F**) Initial attachment of PBCV-1 to a *C*. *variabilis* cell wall. (**G**) Attachment of PBCV-1 to the algal wall and digestion of the wall at the point of attachment. This occurs within 1–3 minutes postinfection (PI) (**H**) Virion particles assemble in defined areas in the cytoplasm named virus assembly centers at ~4 hour PI. Note that both DNA containing (dark centers) and empty capsids. (**I**) A model depicting PBCV-1 assembly into infectious particles including generation of nuclei-derived cisternae decorated with ribosomes (red spheres), which serve as precursors (dark blue) for single bilayer viral membranes (light blue) in the viral assembly centers. (**J**) Localized lysis of cell plasma membrane and cell wall and release of progeny viruses at ~8 hours PI. This figure is modified with permission: Fig 1A–H and J is modified from reference 2 with permission and Fig I is modified from reference 18 with permission.

Chloroviruses are large, icosahedral, dsDNA-containing viruses that replicate in certain unicellular, chlorella-like green algae [1,2]. They exist in freshwater throughout the world with titers as high as thousands of plaque-forming units (PFU) per ml of indigenous water although titers are typically 1–100 PFU/ml. Titers fluctuate during the year with the highest titers typically occurring in the spring and late fall. Known chlorovirus hosts, which are normally symbionts and are often referred to as zoochlorellae, are associated with either the protozoan *Paramecium bursaria* (Fig 1A), the coelenterate *Hydra viridis*, or the heliozoan *Acanthocystis turfacea*. Zoochlorellae are resistant to viruses in their symbiotic state. Fortunately, some zoochlorellae grow independently of their partners in the laboratory, permitting plaque assay of the viruses (Fig 1B) and synchronous infection of their hosts, which allows one to study the virus life cycle in detail.

The genomes of 41 chloroviruses infecting 3 hosts have been sequenced, assembled, and annotated [3]. Collectively the viruses encode genes from 632 protein families; however, any given chlorovirus only has 330 to 416 protein-encoding genes (CDSs). Thus, the genetic diversity among these viruses is huge and many of the proteins are unexpected for a virus. With the exception of homologs solely in other chlorovirus members, about 50% of their CDSs do not match anything in the databases. Some chloroviruses also have introns and inteins, which are rare in viruses.

The prototype chlorovirus *Paramecium bursaria* chlorella virus (PBCV-1) infects *Chlorella variabilis* NC64A, whose 46.2 Mb genome has also been sequenced and annotated [4]. PBCV-1 is an icosahedron (190 nm in diameter) with a spike-like structure at one vertex (Fig 1C) and a few external fibers that extend from some of the capsomers (Fig 1D and 1E) [5]. The outer capsid layer covers a single lipid bilayered membrane, which is required for infection (Fig 1F) [5,6]. The PBCV-1 major capsid protein is a glycoprotein and three of them form a trimeric capsomer, which has pseudo-sixfold symmetry. A proteomic analysis of PBCV-1 virions indicates that the virus has 148 virus-encoded proteins and at least one host-encoded protein [7]. The host protein has similarities to DNA binding proteins.

The PBCV-1 genome is a linear ~331-kb, nonpermuted dsDNA molecule with covalently closed hairpin termini. Identical ~2.2-kb inverted repeats flank each hairpin end. The remainder of the PBCV-1 genome contains primarily single-copy DNA and encodes ~416 proteins [7]. The G+C content of the PBCV-1 genome is ~40%; in contrast, its host nuclear genome is ~67% G+C. PBCV-1 and the other chlorovirus genomes contain methylated bases, which occur in specific DNA sequences [8]. The methylated bases are part of the virus-encoded DNA restriction and DNA modification systems.

## How Do the Chloroviruses Move?

The chloroviruses are typically found in freshwater environments and presumably they move by water currents. However, the chloroviruses associate with the paramecia plasma membrane without infecting the paramecia [9]. Since paramecia swim, this may aid virus movement. In addition, the viruses may move in passive vectors, such as adhering to migratory birds, or move up the food chain and thereby be transported by fish or other aqueous organisms. A recent report indicates that related algal viruses can be transferred in aerosols if their concentrations are high enough [10].

## How Do the Chloroviruses Replicate?

The PBCV-1 virion spike structure first contacts the host cell wall (Fig 1F) and the fibers then aid in attaching the virus to the wall (Fig 1D) (6). PBCV-1 attachment to its host receptor is specific but the nature of the receptor is unknown. The virus spike is too narrow to deliver DNA and likely serves to puncture the wall and then is jettisoned. Following host cell wall degradation by a virus-associated enzyme(s) (Fig 1G), the viral internal membrane presumably fuses with the host membrane, facilitating entry of the viral DNA and virion-associated proteins into the cell, leaving an empty capsid attached to the surface [11]. This fusion process triggers rapid depolarization of the host membrane, probably by a virus encoded K^+^ channel located in the internal membrane of the virus, releasing K^+^ from the cell. The rapid loss of K^+^ and associated water fluxes from the host reduce the host turgor pressure, which aids ejection of viral DNA and virion-associated proteins into the host [12]. Host membrane depolarization also inhibits many host secondary transporters and prevents infection by a second virus.

None of the chloroviruses have a recognizable RNA polymerase gene, so it is assumed that PBCV-1 DNA and viral-associated proteins quickly move to the nucleus where early transcription begins 5 to 10 minutes postinfection (PI) [13]. In this immediate-early phase of infection, host transcription rates decrease [14] and the host transcription machinery is reprogrammed to transcribe viral DNA. Details of reprogramming are unknown but host chromatin remodeling is probably involved because PBCV-1 encodes and packages an enzyme that methylates Lys-27 in histone 3. Circumstantial evidence indicates that this enzyme represses host transcription following PBCV-1 infection [15]. In addition, host chromosomal DNA degradation begins within 5 minutes PI, presumably by PBCV-1 encoded and packaged DNA restriction endonucleases which also inhibit host transcription [16].

The virus dominates the infected cells in the early phase prior to DNA synthesis (0 to 60 minutes PI) as determined by transcriptional profiling. Microarray methods indicate that 63% of the CDSs are expressed early, whereas late phase genes (>60 minutes PI) are expressed until the end of virus replication; 43% of the genes are expressed both early and late [17]. Remarkably, RNA sequencing methods revealed that ~50 CDSs are expressed within the first 7 minutes PI, and by 60 minutes, essentially all of the genes are expressed at some level. At 60 minutes PI, ~40% of the poly (A+)-containing RNAs in the infected cell are PBCV-1 transcripts [13].

At 2 to 3 hours PI, assembly of PBCV-1 capsomers begins in localized regions of the cytoplasm, which become prominent 3 to 4 hours PI (Fig 1H). These localized regions, called virus factories, consist of host cisternae that are derived from the endoplasmic reticulum next to the nuclear envelop (Fig 1I). The cisternae are localized at the periphery of the viral factories and are cleaved into single bilayered membranes [18]. The single bilayered membranes then move to the central region of the virus factories and capsomers form around these membranes leading to the formation of empty virions. Viral DNA is present throughout the host cytoplasm but excluded from the membrane-containing factory sites. DNA packaging occurs in the nascent empty virion particles at the periphery of the virus factories (Fig 1I). Five to 6 hours PI the cytoplasm fills with infectious progeny virus particles and localized lysis of the host cell releases progeny at 6 to 8 hours PI (Fig 1J). Each infected algal cell releases ~1000 particles, of which ~30% form plaques.

## What Are the Consequences of Chlorovirus Infection?

Replication of the chloroviruses results in the death and lysis of the algal cell. Other evolutionarily related algal viruses are partially responsible for terminating massive algal blooms that often occur in marine environments such as brown tides and red tides [e.g., 19]. In addition to terminating algal blooms, the algal viruses have profound effects on aquatic food webs by “shunting” primary production away from higher trophic levels thereby stimulating respiration from the consumption of organic matter liberated from lysed cells.

Several years ago, a research group led by Robert Yolken at Johns Hopkins School of Medicine contacted our laboratory because they had occasionally detected chlorovirus RNA sequences in brain tissues of subjects who had been afflicted with either schizophrenia, bipolar disorder, or major depression [20]. Consequently, we are exploring this unexpected finding with the Yolken laboratory and our first manuscript was published in 2014 [20]. The results can be summarized as follows: Chlorovirus ATCV-1-like DNA sequences, representing a variety of virus genes, were detected in 42 deep throat swabs of 92 apparently normal adults. The presence of the ATCV-1 DNA was associated with a modest but statistically significant decrease in performance on certain cognitive assessments of visual processing and visual motor speed. The effect of ATCV-1 on cognitive behavior was also explored in mice. A one-time gavage inoculation of ATCV-1 infected algae into mice resulted in a subsequent decrease in their performance in certain cognitive traits 6 to 22 weeks later, including ones involving recognition memory and sensory-motor gating. Thirty-six percent of the inoculated mice developed IgG class antibodies to ATCV-1. Finally, a transcriptome analysis conducted on brain tissue (hippocampus) from the mice 22 weeks after gavaging revealed that ~1300 genes were either up- or down-regulated, including many genes in pathways that are relevant to cognitive behavior. A more recent study [21] involved injecting mice intracranially with ATCV-1 and examining behavioral and cognitive tasks 4 weeks later. ATCV-1 infection resulted in impaired delayed location recognition memory, sociability, and reduced anxiety. Additionally, increases in ATCV-1, IL-6, iNOS, IFN-gama, and CD11b expression in the brain was observed at 8 weeks postinfection compared with that in control mice. These results suggest that ATCV-1 infection damages the hippocampus via induction of inflammatory factors.

The mice results prompted a study on the effect of ATCV-1 on murine RAW264.7 macrophages because IL-6, nitric oxide (NO), and ERK MAP-kinase activation in macrophages are linked to cognitive impairments. Approximately 8% of the initial infectious ATCV-1 inoculum persisted in macrophages, and even increased 3 to 4 times within 24 hours PI and then remained fairly constant for at least 72 hours. Moreover, starting at 24 hours, RAW264.7 cells exhibited several cytopathic effects, Annexin V staining and cleaved-caspase 3, consistent with apoptosis [22]. Activation of ERK MAP-kinases occurred in these cells by 30 minutes postinoculation, which preceded expression of IL-6 and NO. Therefore, ATCV-1 persistence in macrophages and induction of inflammatory factors in these macrophages might contribute to declines in cognitive abilities of mice and humans.

## Why Are the Chloroviruses Successful?

Our knowledge of the natural history of the chloroviruses is limited, but they are found in inland watersheds throughout the world and have fluctuations in abundances that reflect the ecological forces controlling their natural hosts. Zoochlorellae are widespread as mutualistic endosymbionts of many protists, as well as basal and higher metazoans. The phylogenetic distribution of the chloroviruses reflects their hosts and should be considered essential members of the symbiotic systems where they are found. But many questions remain unanswered:

1. What supports/activates virus replication in natural conditions?
2. Do the symbiotic hosts continually shed zoochlorellae or are the algae released when the host dies and then become infected?
3. Are there other natural hosts of the chloroviruses and how might one find them?

We continue to search for the answers to these fundamental questions in chlorovirus ecology, especially as it relates to their potential role in human biology.
